# *Ava*II senses m^6^A and inosine sites and enables targeted nanopore direct RNA-sequencing

**DOI:** 10.3389/fmolb.2025.1593637

**Published:** 2025-07-28

**Authors:** Isabel S. Naarmann-De Vries, Tim Preissendörfer, Julian König, Christoph Dieterich

**Affiliations:** ^1^Klaus Tschira Institute for Integrative Computational Cardiology, Department of Internal Medicine III, University Hospital Heidelberg, Heidelberg, Germany; ^2^German Center for Cardiovascular Research (DZHK), Partner Site Heidelberg/Mannheim, Heidelberg, Germany; ^3^ Institute of Molecular Biology (IMB), Mainz, Germany; ^4^Theodor Boveri Institute, Biocenter, University of Würzburg, Würzburg, Germany

**Keywords:** RNA methylation, direct RNA-sequencing, nanopore, DNA endonuclease, RNA modification, RNA cleavage

## Abstract

Nanopore direct RNA-sequencing is the first commercialized method to sequence native RNA directly, thus preserving RNA modifications. With the current technology, sequencing is initiated from the 3′end. While for relatively short polyadenylated RNAs, full coverage is obtained, the 5′end of many long RNAs is not sufficiently covered resulting in a substantial 3′bias. We aimed to cleave such RNAs in a sequence-specific manner in order to generate new unique 3′ends that can be targeted by custom adapters. We identified the DNA endonuclease *Ava*II as a candidate enzyme. *Ava*II was originally described to cleave double-stranded DNA at GGWCC sites, where W is an A or T. Here, we show that *Ava*II cleaves also long RNAs in GGACC contexts, if hybridized to a complementary DNA oligo. Furthermore, we provide evidence that *Ava*II cleavage of RNA is modification sensitive and does not cleave RNA with m^6^A or inosine in the central position. We propose *Ava*II as “methylation sensor” for the *bona fide* DRACH recognition motif GGACC of the m^6^A writer complex. Finally, we show that *Ava*II cleavage products are accessible to targeted Nanopore direct RNA-sequencing.

## 1 Introduction

Nanopore direct RNA-sequencing (DRS) is state-of-the-art to analyze gene expression on isoform level and identify RNA modifications (Diensthuber and Novoa, 2025; Pardo-Palacios et al., 2024). While the method gains increasing importance, it still has technical limitations. One of these limitations is the sequencing start from the 3′end targeting the polyA tail with still limited read length. Here, the limitations may arise either from RNA degradation or failure of the motor protein to processively feed such long molecules through the pore. Consequently, many long cellular RNAs cannot be assessed completely. The current median read length in human whole transcriptome analysis is about 2 kb. On another avenue, one might not be interested in sequencing the whole mRNA transcriptome in specific cases. One possible use case is the analysis of reporter constructs in large screens, such as siRNA screens. Here, it would be advantageous to focus the sequencing capacity on the molecules of interest in multiplexed libraries (Pryszcz et al., 2025; Toorn et al., 2024).

To address these obstacles, we aimed to develop a sequence-specific RNA cleavage tool. The generated products may then be targeted by custom adapters, which can be either specific for a reporter RNA or initiate sequencing in the 5′region of an mRNA. Initial screenings excluded RNase H and the DNA endonuclease *Taq*I for this purpose. While the RNase H generated products with the tested cleavage oligos were too heterogeneous for efficient custom adapter ligation, *Taq*I was only active under conditions that resulted in non-specific RNA degradation (data not shown).

It is a longstanding idea that DNA endonucleases can be “off label” also used to cleave RNA. Usually cleavage is here induced by annealing of a reverse complementary DNA strand (Murray et al., 2010). Among the DNA endonucleases with RNA endonuclease activity, *Ava*II was the most promising candidate. *Ava*II cleaves RNA in the degenerate consensus sequence GGWCC, where W may be an A or T in the case of a DNA substrate (Murray et al., 1976).

Here, we show that *Ava*II cleaves RNA efficiently in GGACC, but not GGUCC or GGICC contexts (I = inosine). GGACC is one of the top m^6^A modified DRACH motifs in humans (Liu et al., 2023). Therefore, we tested if *Ava*II cleavage is dependent on the modification status. We find that *Ava*II cleaves RNA only in unmodified sequences, but not in m^6^A-methylated sites. Thus, *Ava*II can be used as a tool to validate the methylation status of GGACC DRACH motifs. Finally, we show that *Ava*II cleaved RNAs can be targeted with custom adapters for DRS.

## 2 Methods

### 2.1 Generation of *in vitro* transcripts (IVTs)

Generation of the IVTs used in this study has been described previously. IVT1 is the human 18S rRNA (Naarmann-de Vries et al., 2023). IVT2 encodes for Firefly luciferase and has been described previously (Liepelt et al., 2014). Ctrl 2 (Boileau et al., 2021) has been described previously. Ctrl 1 was amplified from pcDNA6 with primers listed in Supplementary Table S2. The templates for all IVTs carry a T7 promoter and were transcribed using the T7 MegaScript kit (Thermo Fisher Scientific) and purified with RNA-Clean and Concentrator-25 kit (Zymo Research). The integrity of the IVTs was analyzed on agarose or TBE urea gels.

### 2.2 Culture of HEK293 cells and isolation of HEK293 total RNA

HEK293 cells were obtained from the DSMZ–German Collection of Microorganisms and Cell Cultures GmbH (ACC 305) and kept in DMEM (Thermo Fisher Scientific) supplemented with 10% fetal bovine serum (Merck) and 1x penicillin/streptomycin (Thermo Fisher Scientific) at 37°C, 5% CO_2_. RNA was isolated with Trizol (Thermo Fisher Scientific) according to the manufacturer’s instructions. Purity and integrity of the isolated RNA were assessed by Nanodrop (Thermo Fisher Scientific) and on Fragment Analyzer (Agilent) measurements.

### 2.3 Titration of *Ava*II magnesium concentration

The magnesium requirement of *Ava*II was assessed by cleavage of a DNA substrate (pXR003, Addgene #109053). 500 ng plasmid were digested in a total volume of 20 µL with 1 µL *Ava*II (New England Biolabs) in different buffers for 15 min at 37°C. A control reaction in the provided rCutSmart buffer was performed in parallel. The other reactions were incubated in 50 mM NaCl, 20 mM Tris pH 8.0 (basic buffer) supplemented with 0.5 mM–10 mM MgCl_2_ as indicated. Reactions were analyzed on a 1% agarose gel.

### 2.4 *Ava*II RNA cleavage of IVTs

**FIGURE 1:**
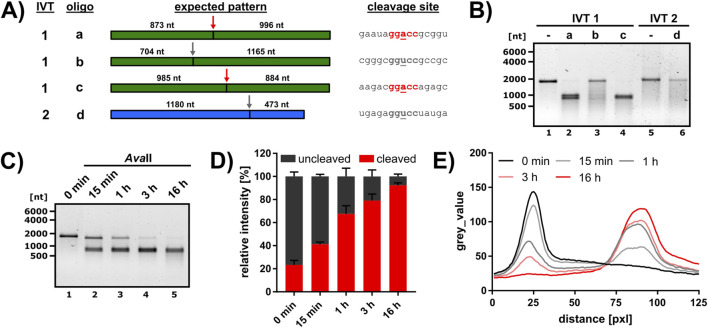
Test of *Ava*II cleavage in different sequence contexts and optimization of cleavage conditions. **(A)** Cleavage of RNA by *Ava*II was analyzed employing two IVTs (IVT1 = green, IVT2 = blue) and four DNA oligos (a-d) for targeting. The *Ava*II cleavage site is indicated by an arrow (red = central A, gray = central U) and the size of the expected cleavage products is given. The RNA sequence at the cleavage site is depicted on the right, the sequence of the corresponding cleavage oligo is listed in Supplementary Table S1. The recognition site is marked in bold, and the central variable nucleotide underlined. **(B)** IVT1 and IVT2 were annealed to the respective cleavage oligo and subjected to *Ava*II cleavage (16 h reaction time). Reaction products were analyzed on a 1% agarose gel. The quantitative analysis of oligo a and b cleavage is presented in Supplementary Figures S2A–C. **(C–E)** Time course of IVT1 cleavage with *Ava*II and oligo a. **(C)** Representative time course analyzed on a 1% agarose gel. **(D)** The band intensities of the uncleaved IVT 1 (dark gray) and the cleavage products (red) were quantified and normalized to ctrl 1 and ctrl 2 (see Supplementary Material) and expressed as relative intensity, where the sum of cleaved and uncleaved was defined at 100% (n = 3). **(E)** Mean of lane profiles for IVT1 and the cleavage products (n = 3).

1 µg IVT was annealed to 10 pmol cleavage oligo (Supplementary Table S1) in 10 mM Tris pH 7.5, 50 mM NaCl in a total volume of 20 µL (2 min, 95°C; 0.1 °C/s to 22°C; 5 min, 22°C). Reactions were then adjusted to 50 µL total volume with basic buffer supplemented with 0.5 mM MgCl_2_ and 1 µL *Ava*II. If not otherwise indicated, reactions were incubated overnight at 37°C. Reactions were cleaned up by addition of 1.8x RNA Clean XP beads (Beckman Coulter) (incubation 15 min on ice). After collecting the beads on a magnetic rack, beads were washed two times with 80% ethanol and eluted in 10 µL H_2_O. Reactions were analyzed on 1% agarose gels. For the experiment in Supplementary Figure S2 and the time course (Figures 1D,E), 250 ng ctrl 2 (80 nt) were added to the annealing step and 50 ng ctrl 1 (400 nt) were added directly before the addition of the RNA Clean XP beads. The uncleaved RNA was incubated overnight at 37°C as well. Band intensities of full length IVT1, ctrl 1 and ctrl 2 were measured with ImageJ. The relative amount of full length IVT2 was calculated normalized to ctrl 1+ctrl 2. Lane profiles were received from ImageJ.

### 2.5 *Ava*II cleavage in HEK293 total RNA

**FIGURE 2:**
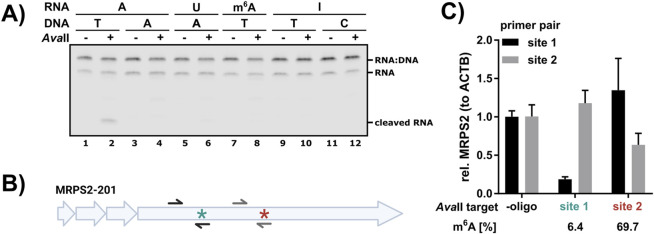
*Ava*II cleavage is dependent on the modification status. **(A)** 5′Cy5.5-labeled RNA oligos with an *Ava*II recognition site bearing different central nucleotides as indicated were subjected to *Ava*II cleavage with complementary DNA oligo bearing different central nucleotides in the *Ava*II recognition motif. Cleavage reactions were analyzed on 15% TBE urea gels. Replicate analysis and quantification are presented in Supplementary Figure S3. **(B)** Schematic representation of the MRPS2-201 transcript. The lowly methylated m^6^A site 1 is marked with a turquoise star and the highly methylated m^6^A site 2 with a dark red star. Both m^6^A sites are in GGACC contexts. See Supplementary Table S1 for the reverse complementary *Ava*II cleavage oligos. Integrity of the respective sites was detected by RT-qPCR analysis **(C)** with a forward primer binding upstream of the m^6^A site and a reverse primer covering the cleavage site (black = site 1, gray = site 2). **(B)** Two GGACC sites in MRPS2 in HEK293 RNA were targeted with *Ava*II. According to GLORI and DRS analysis, site 1 (turquoise) is lowly methylated, whereas site 2 (dark red) is highly methylated. Cleavage efficiency for each targeted site was assessed by RT-qPCR analysis.

2 µg total RNA was annealed to 100 pmol cleavage oligo (Supplementary Table S1) in 10 mM Tris pH 7.5, 50 mM NaCl in a total volume of 20 µL (2 min, 95°C; 0.1 °C/s to 22°C; 5 min, 22°C). Reactions were then adjusted to 50 µL total volume with basic buffer supplemented with 0.5 mM MgCl_2_ and 2 µL *Ava*II. Reactions were incubated overnight at 37°C and cleaned up as described above. Identical RNA volumes were reverse transcribed with Maxima H Minus cDNA synthesis kit (Thermo Fisher Scientific) and Random primers. Regions covering the targeted m^6^A cleavage sites (see Figure 2B) were amplified by RT-qPCR using PowerUp SYBRgreen master mix (Thermo Fisher Scientific) on a ViiA7 instrument (Thermo Fisher Scientific). Levels were normalized to ACTB and a control reaction without cleavage oligo. All qPCR primers are listed in Supplementary Table S2.

### 2.6 Nanopore direct RNA-sequencing of *Ava*II cleaved RNA

IVT1 was cleaved with oligo a as described above. The library was prepared with two custom adapters as described in (Naarmann-de Vries et al., 2023) using the SQK-RNA002 kit and sequenced on a Flongle flow cell (FLO-FLG001). Custom adapter 1 targets the 3′end (Naarmann-de Vries et al., 2023) and custom adapter 2 targets the *Ava*II generated end (sequence specific oligo B: GAGGCGAGCGGTCAATTTTCCTAAGAGCAAGAAGAAGCCCTATTCCATT). Basecalling was performed with Guppy 6.5.7 in High-accuracy mode (HAC) and data were aligned to the human 18S rRNA reference (NR_003286.4) with minimap2. Data were visualized in Integrative Genomics Viewer (IGV).

### 2.7 RNA oligo cleavage assay

RNA oligos with a length of 48 nts and a 5′Cy5.5 fluorophore were purchased from GenScript. The basic sequence with a GGACC *Ava*II recognition motif was derived from (Murray et al., 2010). In additional oligos, the central nucleotide was replaced by U, m^6^A and inosine. All RNA oligos are listed in Supplementary Table S3. 20 pmol RNA oligo was combined with 20 pmol DNA oligo (Supplementary Table S1) as indicated and incubated in 1x rCutSmart buffer (New England Biolabs) with 1 µL *Ava*II overnight at 37°C in a total volume of 50 µL. 10 μL of the reaction was combined with 1 Vol TBE-urea sample buffer (Thermo Fisher Scientific) and denatured (10 min, 65°C; 2 min, ice). Samples were loaded to a 15% Novex TBE urea PAGE and monitored at a Licor Odyssey XF instrument (LI-COR Biosciences).

## 3 Results

### 3.1 *Ava*II can be directed to cleave RNA in GGACC sequence contexts

We selected the DNA endonuclease *Ava*II, as its capacity to cleave RNA was shown previously (Jo et al., 2022; Murray et al., 2010). Standard DNA endonuclease reactions contain high concentrations of divalent cations, more specifically magnesium. For instance, the rCutSmart buffer provided by New England Biolabs contains 10 mM magnesium acetate. Such high magnesium concentrations cause rapid degradation of RNA, especially at elevated temperatures, which is widely used to fragment RNA for short-read sequencing approaches. To avoid such reaction conditions and obtain intact RNA suitable for DRS, we first optimized the *Ava*II reaction buffer to reduce the magnesium concentration and subsequently non-specific RNA degradation. With a classical plasmid as DNA substrate, we found that the cleavage of DNA with 0.5 mM MgCl_2_ was as efficient as with 10 mM MgCl_2_ (Supplementary Figure S1) and used a buffer with 0.5 mM MgCl_2_ throughout.

So far, the capacity of *Ava*II to cleave RNAs has been mainly tested on artificial substrates (Jo et al., 2022; Kisiala et al., 2020; Murray et al., 2010). We initially assessed *Ava*II cleavage on natural sequences within two GGACC and two GGUCC sequence contexts using *in vitro* transcribed RNAs (IVT1 and IVT2, see Methods). For every cleavage site, a reverse complementary DNA oligos with a length of 40 nucleotides was annealed to the respective RNA. The IVT-oligo combinations, expected cleavage products and sequence contexts at the cleavage site are shown in Figure 1A. All cleavage reactions, including an uncleaved control (IVT in H_2_O, same incubation time and temperature), were analyzed on agarose gels. Interestingly, we detected efficient *Ava*II cleavage in GGACC sequence contexts, but not GGUCC sequence contexts (Figure 1B). We noticed however some non-specific degradation in the presence of the GGUCC oligos. To exclude that this degradation is induced by insufficient probe specificity, we performed a quantitative analysis for oligos a and b, and also included a control reaction without DNA oligo (Supplementary Figure S2A). This analysis revealed a certain extent of non-specific RNA decay, presumably induced by the magnesium in the reaction (Supplementary Figures S2B, C), however no insufficient probe specificity. We analyzed whether shorter reaction times could improve increase RNA integrity, but a time course experiment using IVT1 and oligo a revealed that the most efficient cleavage was detected after overnight incubation (Figures 1C–E). We noticed a minor fraction of 7.6% IVT1 that was resistant to *Ava*II cleavage (Figure 1D). However, neither an increase in the amount of DNA oligo nor enzyme further improved cleavage (data not shown).

### 3.2 *Ava*II cleavage is modification sensitive

As GGACC is one of the most abundantly m^6^A methylated DRACH motifs, and uridine is not tolerated as central nucleotide, we speculated that *Ava*II cleavage of RNA might be modification sensitive and in particular m^6^A methylation sensitive. To test this hypothesis, 5′Cy5.5-labeled RNA oligos of 48 nucleotides length with different central nucleotides in the GGxCC *Ava*II recognition motif were digested with different complementary DNA oligos (Figure 2A). As expected, we detected significant cleavage with the pair A(RNA):T(DNA) (Figure 2A; Supplementary Figure S3). The presence of either m^6^A or inosine blocked *Ava*II cleavage, comparable to U (Figure 2A; Supplementary Figure S3).

As this artificial test system does not reflect the abundance and level of endogenous m^6^A sites, we analyzed *Ava*II cleavage at endogenous target sites in HEK293 cells. Based on DRS analysis employing mAFiA (Chan et al., 2024) and glyoxal and nitrite-mediated deamination of unmethylated adenosines (GLORI) (Liu et al., 2023), we selected MRPS2 as target, which has two m^6^A sites in a GGACC context: site 1 is lowly methylated (6.4% according to mAFiA) and site 2 is highly methylated (69.7% according to mAFiA) (Chan et al., 2024; Figure 2B). We designed cleavage oligos for both sites and set up three reactions: “minus oligo”, “site 1 oligo” and “site 2 oligo”. The integrity of the targeted site was checked in RT-qPCR with primers spanning the respective region. This analysis revealed that site 1 was efficiently cleaved by “site 1 oligo”, but not “site 2 oligo”. On the other hand, site 2 oligo induced only moderate cleavage at site 2 and no cleavage at site 1. Overall, this analysis revealed a good correlation (Supplementary Table S4) between methylation level determined by other methods and non-cleaved target (Figure 2C). Nonspecific cleavage by site 1 oligo at site 2 and vice versa is not detected. Based on this observation we propose *Ava*II as modification, and especially m^6^A methylation sensor in GGACC contexts in cellular RNA.

### 3.3 Cleavage products can be targeted for DRS

**FIGURE 3:**
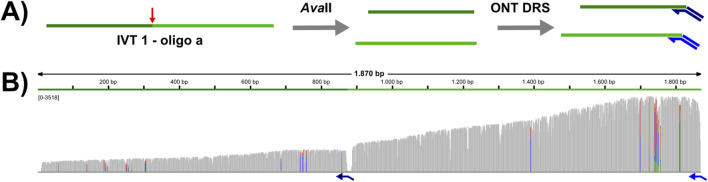
*Ava*II cleaved RNAs can be targeted for DRS with custom adapters. **(A)** Experimental design: IVT1 was subjected to *Ava*II cleavage with oligo a. Purified RNA was used to prepare a DRS library with two custom adapters (indicated in dark and mid blue). Adapter 1 (mid blue) targets the natural 3′end, whereas adapter 2 (dark blue) targets the newly generated end. **(B)** Visualization of the DRS data with IGV. The coverage track of all reads aligning to the IVT1 reference sequence is shown. Gray bars represent positions that match the reference sequence, mismatched positions are shown colored (green = A, red = T, blue = C, orange = G, allele frequency threshold = 0.2).

Selective direct RNA sequencing of the generated cleavage products (for example a 5′fragment of a long cellular RNA) is only possible after ligation of a sequence specific adapter. T4 DNA ligase, which is used for this ligation requires a nicked template and does not tolerate gaps or flaps. Thus, an exactly defined cleavage site is required to design the appropriate custom adapter. Our initial screens excluded broadly used RNase H and the DNA endonuclease *Taq*I for this task (data not shown). As a proof-of-principle, we designed a custom adapter for IVT1 cleaved in presence of DNA oligo a (see Figure 1), and subjected the cleavage products to DRS library preparation, with two custom adapters: the first adapter targets the natural 3′end, whereas the second adapter targets the newly generated 3′end (Figure 3A). This test revealed that *Ava*II cleaved RNAs can be sequenced specifically by DRS (Figure 3B), which opens up novel opportunities in design of DRS experiments.

## 4 Discussion

In this work, we present the DNA endonuclease *Ava*II as modification and especially m^6^A methylation sensor. Directed by an antisense DNA oligonucleotide, *Ava*II specifically cleaves non-modified GGACC sites in a complex mixture of cellular RNAs. m^6^A sites in this context are not cleaved. Thus, *Ava*II cleavage can be applied as an orthogonal method for validation of m^6^A sites identified by other (high throughput) methods. We are aware of the limitations of the current method, as cleavage is limited to only one of 18 DRACH motifs and inhibited by the presence of inosine as well. We propose the screening of other DNA endonucleases to identify more enzymes that cleave RNA dependent on the modification status. Furthermore, the sequence specificity of *Ava*II may be changed to other DRACH motifs by protein engineering approaches as directed evolution.

As a second use case, we show that *Ava*II cleaved RNAs can be targeted specifically for DRS with custom adapters. Targeted DRS requires an exactly defined 3′end. Preliminary analysis excluded RNase H due to generation of heterogeneous ends and *Taq*I, because the reaction conditions were incompatible with preserving RNA integrity. The proof-of-principle analysis shown here, enables for example the sequencing of 5′fragments of long cellular RNAs or the targeted sequencing of polyadenylated reporter RNAs. In the latter case, the polyA tail will be removed to generate a unique 3′end. Here, the specificity of *Ava*II cleavage is notable, as only sites annealed to a complementary DNA oligo will be cleaved. On the other hand, the *Ava*II recognition sequence GGWCC may not be available in all cases, potentially limiting the application. Furthermore, we advise to first check the m^6^A status of recognition motifs.

The *Ava*II cleavage approach presented here, is impacted by the relatively low activity of *Ava*II on DNA–RNA substrates compared to the cleavage of pure DNA substrates (Murray et al., 2010). This results in a minor fraction of uncleaved RNA and the requirement of long incubation times, which may negatively impact RNA integrity. Thus, we see *Ava*II as the first step in developing a toolkit for site specific RNA cleavage, which will hopefully be extended by additional RNA endonucleases in the future.

In summary, we present two methods that extend the toolkit of RNA researchers, interested in RNA modifications and long-read sequencing. Further work will broaden the applicability of the proposed methodology.

## Data Availability

The data presented in the study are deposited in the Zenodo repository at https://doi.org/10.5281/zenodo.16036643.
