# Supplementary material for: AvaII senses m6A and inosine sites and enables targeted nanopore direct RNA-sequencing
Source: Front Mol Biosci. 2025 Jul 28;12:1593637. doi: 10.3389/fmolb.2025.1593637 (PMC12336230; doi:10.3389/fmolb.2025.1593637)

Supplementary Material

# Supplementary Figures and Tables

## Supplementary Figures


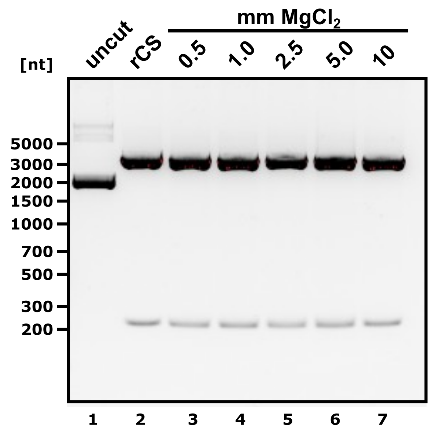


**Supplementary Figure 1.** Titration of magnesium concentration for *Ava*II cleavage. A DNA substrate (plasmid pXR003) was cleaved with *Ava*II under the indicated reaction conditions and analyzed on a 1% agarose gel. rCS = rCutSmart buffer provided by New England Biolabs, with 10 mM magnesium acetate. Size of the expected cleavage products: 2681 bp and 222 bp. GeneRuler 1kb+ ladder (Thermo Fisher Scientific) was used as size standard.


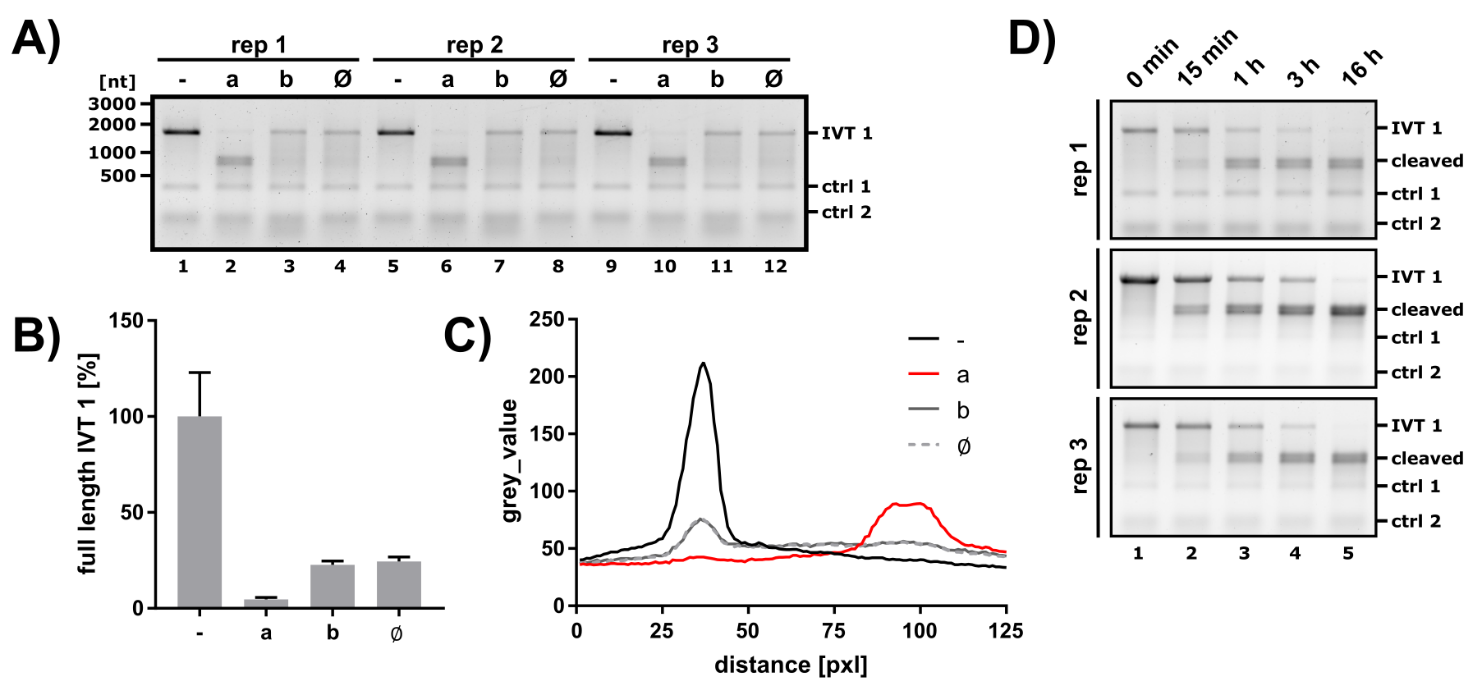


**Supplementary Figure 2.** Quantitative analysis of *Ava*II RNA cleavage. A) IVT1 was subjected to *Ava*II cleavage with oligo a (lanes 2, 6, 10), oligo b (lanes 3, 7, 11) or without cleavage oligo (∅, lanes 4, 8, 12) for 16h at 37°C. Uncut IVT1 in nuclease free water was incubated in parallel (lanes 1, 5, 9). To all reactions, 50 ng of ctrl 2 was added. Prior to SPRI beads clean up, ctrl 1 was added to all reactions. Same volumes were analyzed on a 1% TAE agarose gel. B) Quantification of full length IVT 1, normalized to ctrl 1 and ctrl 2. The mean and standard deviation (n = 3) is shown. C) Mean of lane profiles for IVT1 and the cleavage products (n = 3). Bands and lanes were quantified with ImageJ. D) Agarose gels used for quantitative analysis of the time course presented in Figure 1 D and E.


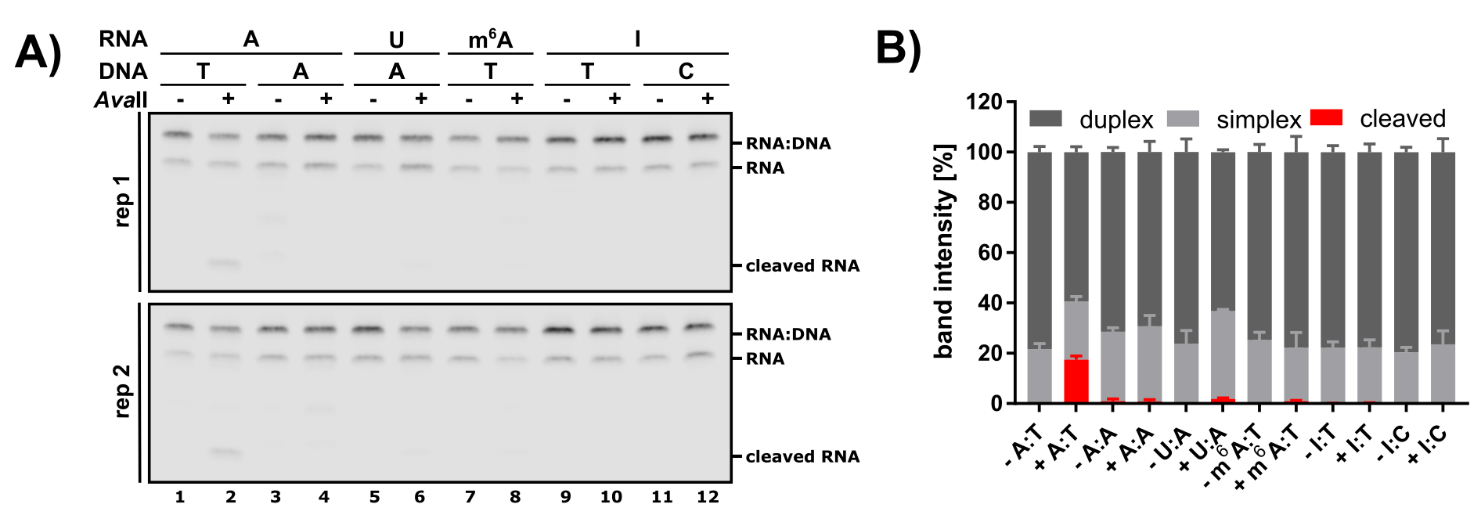


**Supplementary Figure 3.** *Ava*II cleavage site specificity. A) Replicate analysis of the RNA oligo cleavage shown in Figure 2A (replicate 3). Here, the analysis of replicates 1 and 2 is shown. B) Quantitative analysis of the RNA oligo cleavage assay. The band intensities of duplex (annealed to the DNA oligo), simplex RNA oligo and the cleavage product were determined with ImageJ and expressed as percent of total (n =3). Significant cleavage was only detectable for the A(RNA):T(DNA) duplex (p < 0.001). 2-way ANOVA with Tukey’s multiple comparisons test. Only the respective – enzyme vs. + enzyme comparisons were considered. For U(RNA):A(DNA) the relative fraction of duplex was significantly reduced in the presence of *Ava*II (p < 0.001), but no cleavage was detectable.

## Supplementary Tables

| **Name** | **Sequence** | **position** |
| --- | --- | --- |
| IVT1_a | AACAAAATAGAACCGCGGTCCTATTCCATTATTCCTAGCT | 873 |
| IVT1_b | TGGCTCGCCTCGCGGCGGACCGCCCGCCCGCTCCCAAGAT | 704 |
| IVT1_c | CAAATGCTTTCGCTCTGGTCCGTCTTGCGCCGGTCCAAGA | 970 |
| IVT2_d | ACCGGACATAATCATAGGACCTCTCACACACAGTTCGCCT | 1180 |
| IM398D-T | CGTGAATAGTTTCCTGGATTCGAGGTCCTAGGTACCTATTAATTTTCG |  |
| IM398D-A | CGTGAATAGTTTCCTGGATTCGAGGACCTAGGTACCTATTAATTTTCG |  |
| IM398D-C | CGTGAATAGTTTCCTGGATTCGAGGCCCTAGGTACCTATTAATTTTCG |  |
| MRPS2_1 | TGTGCAGGAAGATGATGAGGTCCGGCAGGCGGACCGTGGG | Chr9:135503815 |
| MRPS2_2 | GAGGGTGGGCTGGCCCCTGGTCCCCGGGCTCCTTCTGGCC | Chr9:135504085 |

**Supplementary Table 1**. *Ava*II DNA cleavage oligos. The positions indicate the cleavage position in the nucleotide sequence of the IVTs or the genomic position for MRPS2.

| **Name** | **sequence** |
| --- | --- |
| MRPS2_1_ctrl fw | CCCACACTCGCTACTTCAG |
| MRPS2_1_ctrl rv | CACGTGTGGCTCAAAGATG |
| MRPS2_2_ctrl fw | GCAGGTTGAGGCTCTCTATC |
| MRPS2_2_ctrl rv | GGGAGGAGAGTGAACATCAC |
| ACTB fw | TCCCTGGAGAAGAGCTACG |
| ACTB rv | GTAGTTTCGTGGATGCCACA |
| T7 Ctrl 1 fw | TAATACGACTCACTATAGATGGCCAAGCCTTTGTCTC |
| Ctrl 1 rv | TTAGCCCTCCCACACATAAC |

**Supplementary Table 2.** PCR primers used in this study

| **Name** | **Sequence** |
| --- | --- |
| IM399R_A | /5'CY5.5_N/CGAAAAUUAAUAGGUACCUAGGACCUCGAAUCCAGGAAACUAUUCACG |
| IM399R_U | /5'CY5.5_N/CGAAAAUUAAUAGGUACCUAGGUCCUCGAAUCCAGGAAACUAUUCACG |
| IM399R_I | /5'CY5.5_N/CGAAAAUUAAUAGGUACCUAGG/irI/CCUCGAAUCCAGGAAACUAUUCACG |
| IM399R_m6A | /5'CY5.5_N/CGAAAAUUAAUAGGUACCUAGG/iN6-Me-rA/CCUCGAAUCCAGGAAACUAUUCACG |

**Supplementary Table 3.** RNA oligos for *Ava*II cleavage assays.

| **Method** | **Site 1** | **Site 2** |
| --- | --- | --- |
| mAFiA | 6.4% | 69.7% |
| GLORI | < 10% | 86.5% |
| *Ava*II | 18.8% | 63.8% |

**Supplementary Table 4.** m^6^A levels/ *Ava*II resistant RNA detected for two GGACC sites in MRPS2 with different methods.

**Supplementary Material**: Uncropped Gel Images. The regions presented in the Figures are marked.

Figure 1B:


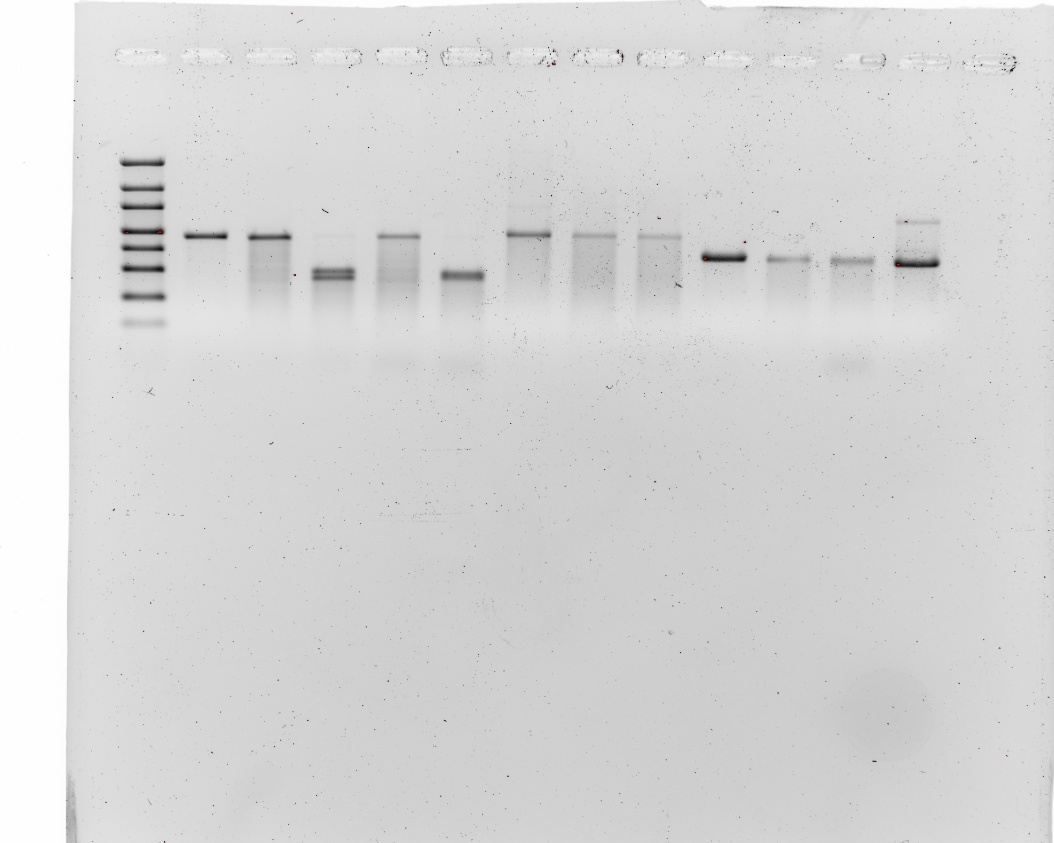


Figure 1C:


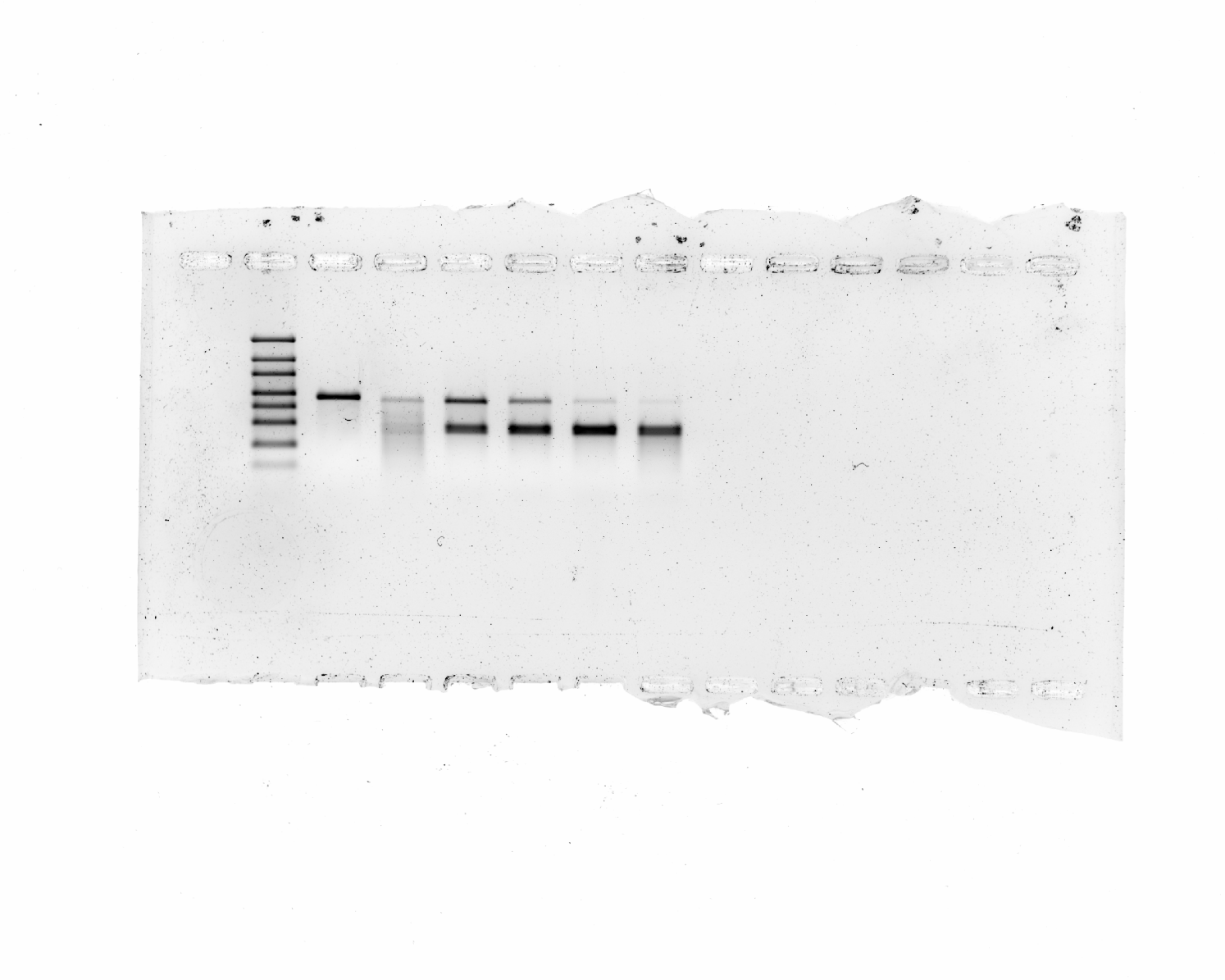


Figure 2A:


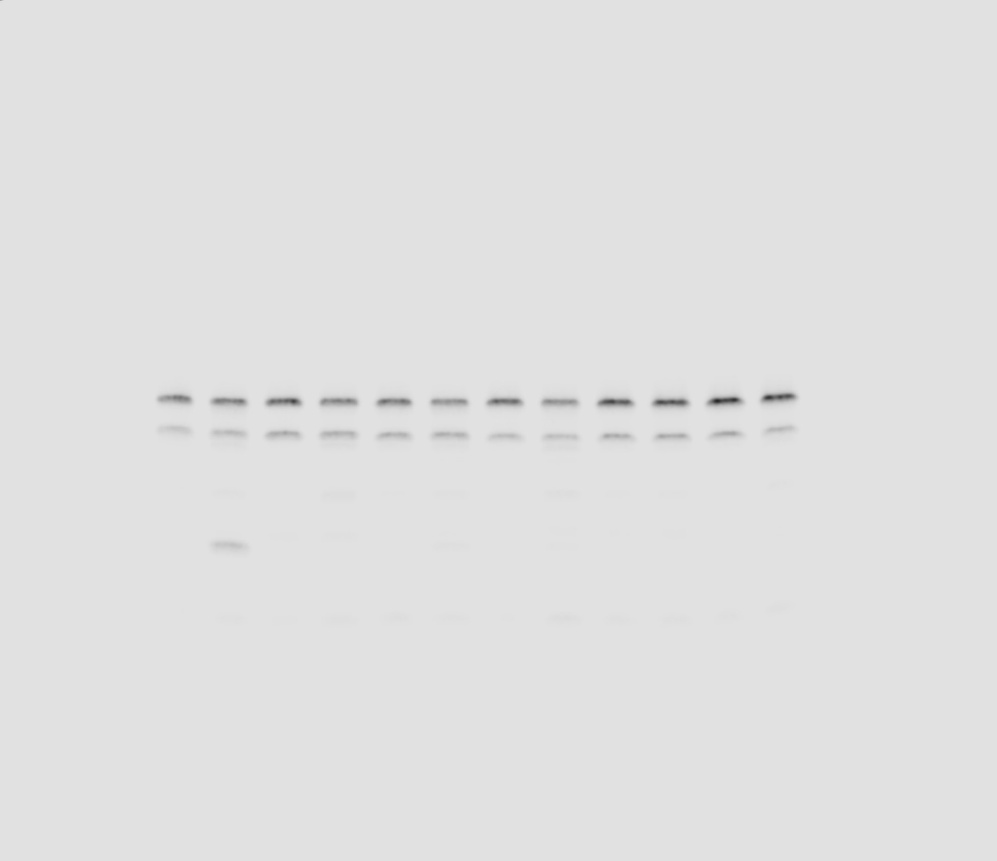


Figure S1:


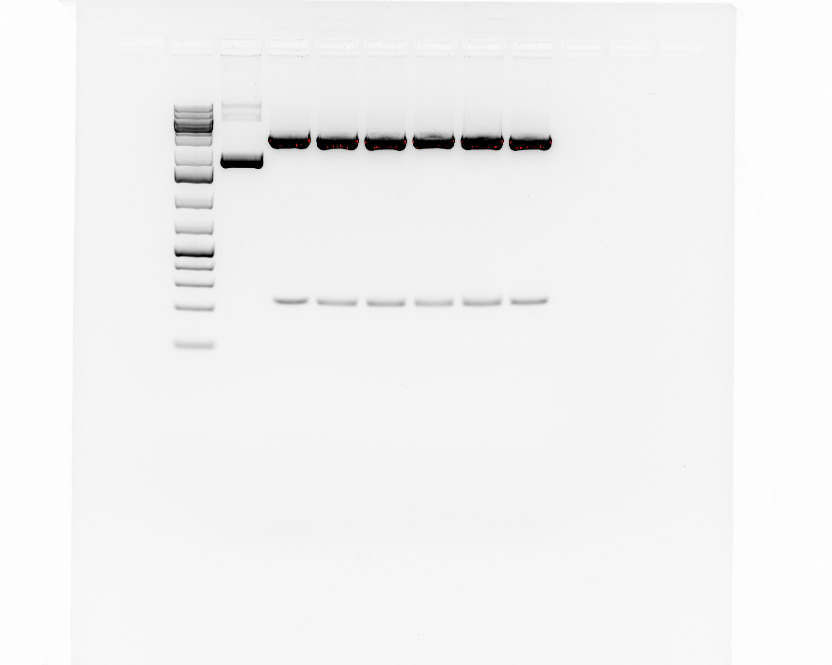


Figure S2A:


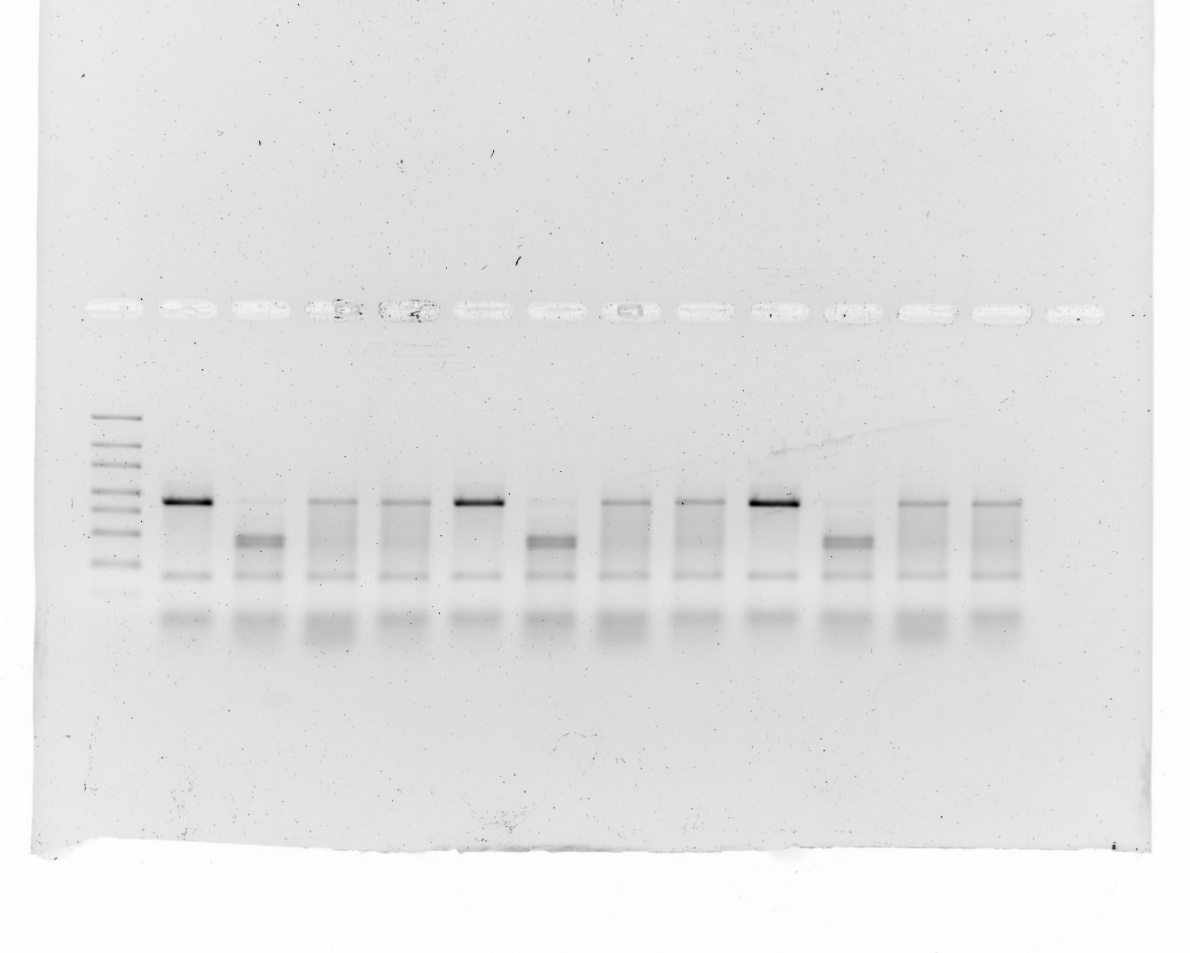


Figure S2D replicate 1:


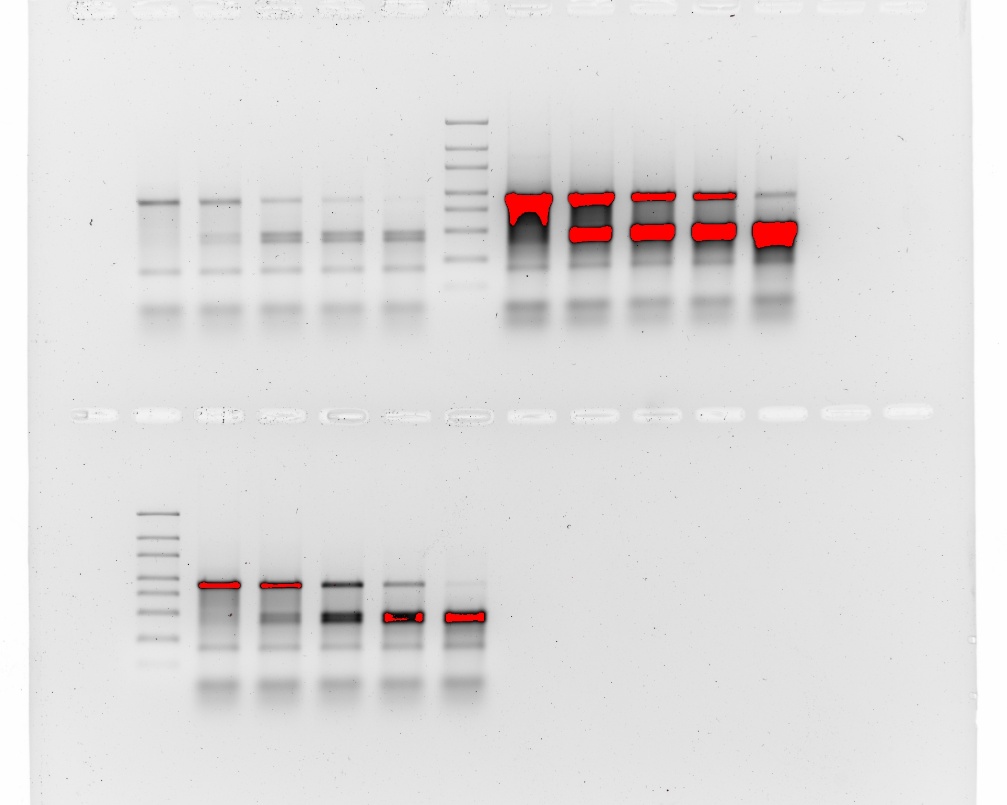


Figure S2 D replicate 2:


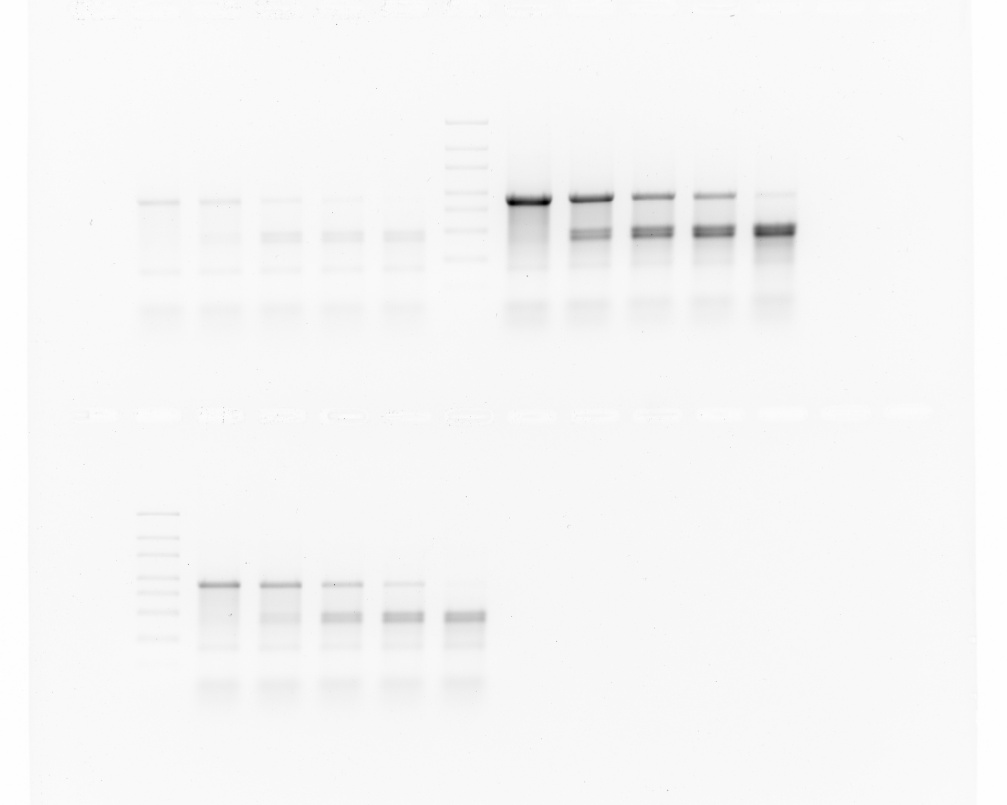


Figure S2D, replicate 3


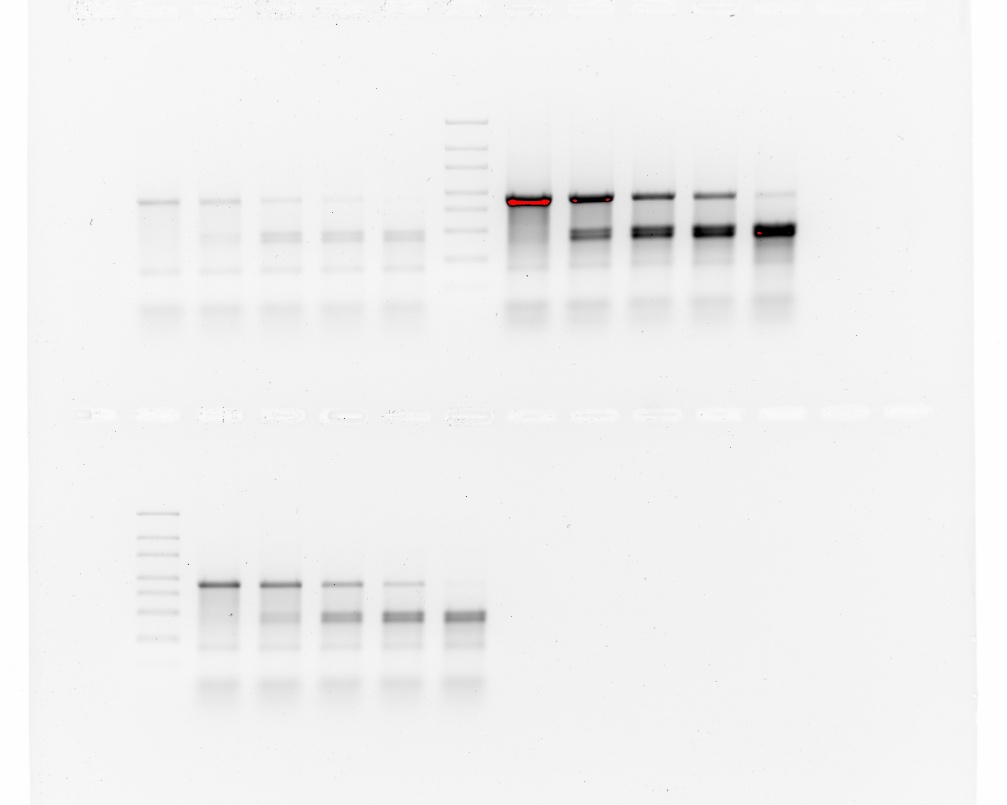


Figure S3A, replicate 1:


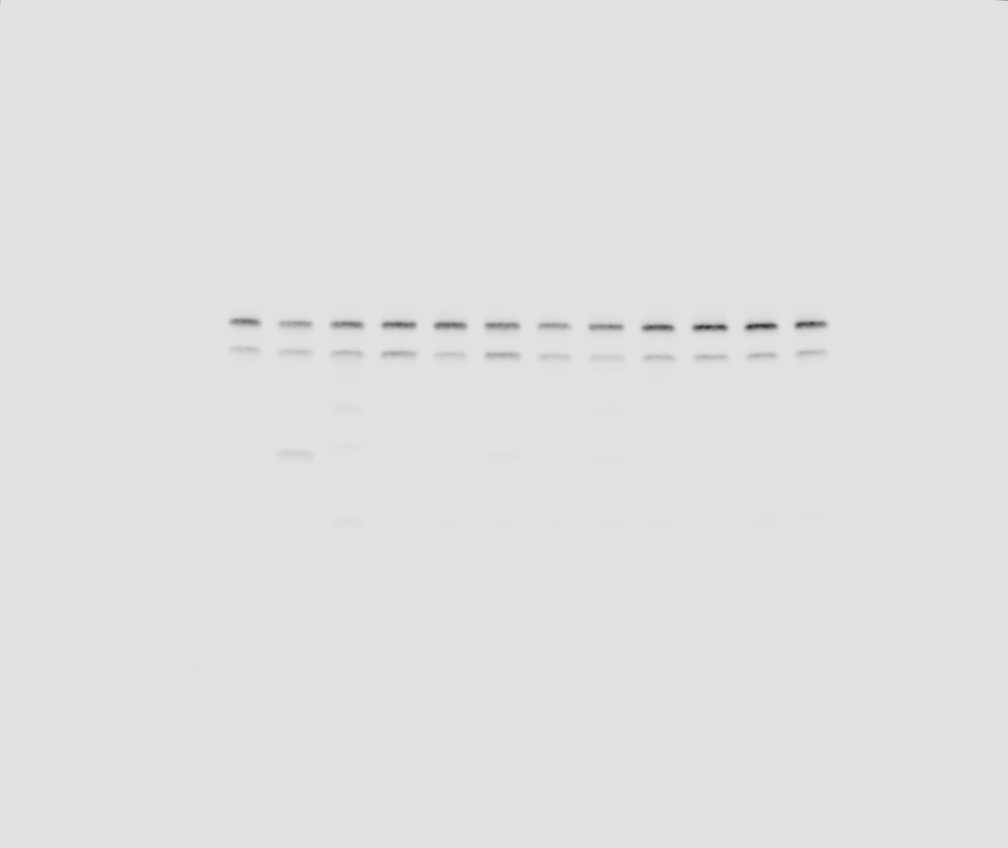


Figure S3A, replicate 2:


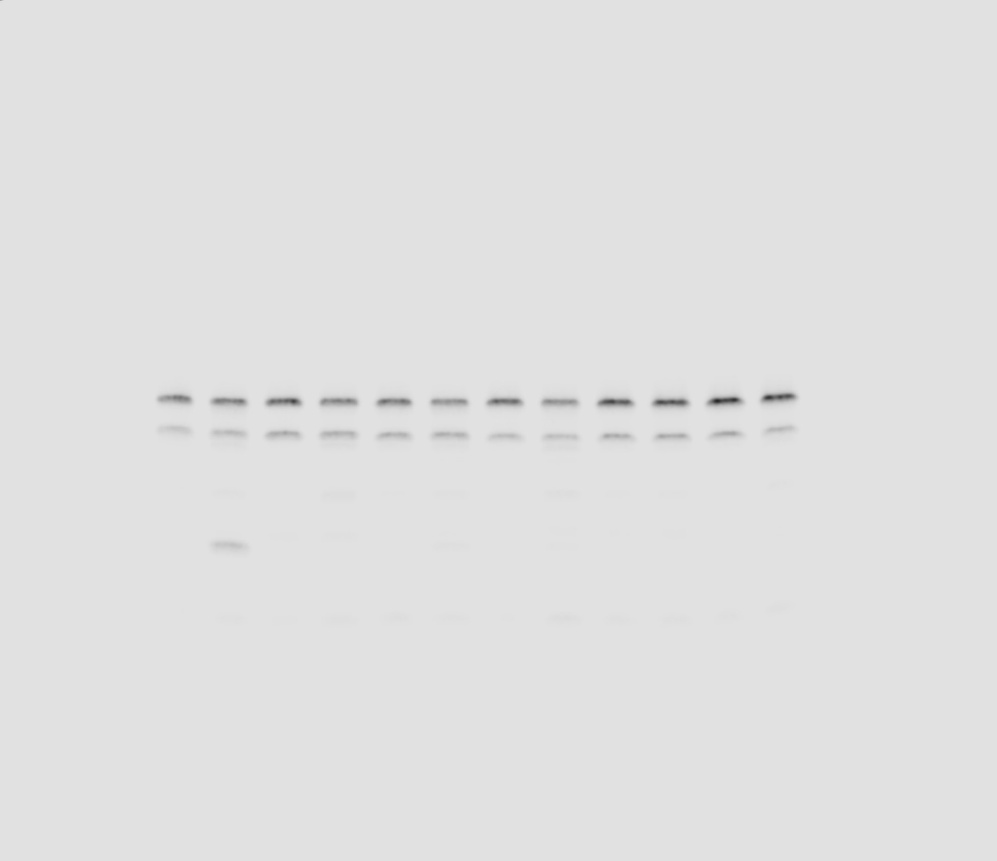

Supplement: Supplementary file 1 [file Supplementaryfile1.docx]
